# Pathological Analysis of Medial and Intimal Calcification in Lower Extremity Artery Disease: Impact of Hemodialysis

**DOI:** 10.1016/j.jacadv.2023.100656

**Published:** 2023-10-19

**Authors:** Tsukasa Kato, Sho Torii, Norihito Nakamura, Kazuki Aihara, Yuta Terabe, Osamu Iida, Takahiro Tokuda, Tatsuya Nakama, Yo Kawahara, Junichi Miyamoto, Takafumi Saito, Norihiko Kamioka, Tsutomu Murakami, Takeshi Ijichi, Makoto Natsumeda, Shigemitsu Tanaka, Yohei Ohno, Gaku Nakazawa, Hiroyuki Watanabe, Yuji Ikari

**Affiliations:** a Department of Cardiology, Akita University School of Medicine, Akita, Japan; b Department of Cardiology, Tokai University School of Medicine, Isehara, Japan; c Kasukabe Chuo General Hospital, Limb Salvage Center, Kasukabe, Japan; d Kansai Rosai Hospital, Cardiovascular Center, Amagasaki, Japan; e Department of Cardiology, Nagoya Heart Center, Nagoya, Japan; f Department of Cardiology, Tokyo Bay Medical Center, Urayasu, Japan; g Division of Vascular Surgery, Department of Surgery, Jikei University School of Medicine, Tokyo, Japan; h Department of Cardiology, Isehara Kyodo Hospital, Isehara, Japan; i Kindai University Faculty of Medicine, Department of Cardiology, Osaka, Japan

**Keywords:** atherosclerosis, chronic limb-threatening ischemia, hemodialysis, intimal calcification, lower extremity artery disease, medial calcification

## Abstract

**Background:**

The prevalence and degree of lower extremity artery disease in hemodialysis (HD) patients is higher than in the general population. However, the pathological features have not yet been evaluated.

**Objectives:**

The aim of the study was: 1) to compare lesion characteristics of lower extremity artery disease in HD vs non-HD patients; and 2) to determine factors associated with severe medial calcification.

**Methods:**

Seventy-seven lower limb arteries were assessed from 36 patients (median age 77 years; 23 men; 21 HD and 15 non-HD) who underwent autopsy or lower limb amputation. Arteries were serially cut at 3- to 4-mm intervals creating 2,319 histological sections. Morphometric analysis and calcification measurements were performed using ZEN software. Calcification with a circumferential angle (arc) ≥180° was defined as severe calcification. Multivariable logistic regression was used to identify risk factors for severe medial calcification.

**Results:**

The degree of the medial calcification arc was significantly higher in the HD group compared to the non-HD group (*P* < 0.0001). In the multivariable analysis, HD was associated with severe medial calcification in below-the-knee lesions (OR: 17.1; *P* = 0.02). The degree of intimal calcification in above-the-knee lesions was also significantly higher in HD patients with a higher prevalence of advanced atherosclerotic plaque (*P* = 0.02). The prevalence of severe bone formation was more common in the HD patients (*P* = 0.01).

**Conclusions:**

Hemodialysis patients demonstrated a higher degree of medial and intimal calcification compared with non-HD patients. The difference was more prominent in the medial calcification of below-the-knee lesions.

The latest systematic review by Fowkes et al^1^ reports that there are more than 200 million people worldwide with lower extremity artery disease (LEAD). The LEAD prevalence in the general population is reported to be between 1.4% and 8.3%,^1^^,^^2^ whereas in hemodialysis (HD) patients it is significantly higher at 25.3%.^3^ HD patients with LEAD also have a worse prognosis after revascularization such as endovascular therapy and bypass.^4^, ^5^, ^6^ The reasons for the poor clinical outcome in patients undergoing HD have not been well evaluated. Losurdo et al^7^ reported that medial calcification of below-the-knee (BK) arteries is strongly associated with the risk of lower extremity amputation in patients with diabetes. Another study also demonstrated higher LEAD prevalence and severity in HD patients with higher calcification scores in lower extremity arteries. However, previous studies, have based the diagnosis of medial or intimal calcification on X-rays^8^^,^^9^ or computed tomography (CT),^10^^,^^11^ which may not be precise enough for calcification localization.

Detailed pathological analysis of atherosclerosis progression in coronary arteries has played an essential role in preventing atherosclerosis and developing percutaneous coronary interventions, such as balloon angioplasty, bare metal stents, and drug-eluting stents.^12^, ^13^, ^14^, ^15^ Previous pathological LEAD analysis has been done in patients with abundant risk factors^16^ and chronic limb-threatening ischemia (CLTI).^17^ However, there are no systematic evaluations of the pathologic features of lower extremity arteries in HD patients with LEAD. Such analysis could reveal the characteristics of the diseased peripheral arteries, facilitating better strategies to prevent atherosclerosis progression in HD patients, for example in improving the quality of dialysis, which depends upon dialysis membranes and fluids. Additionally, a better understanding of the pathological features would promote the development of newer devices for endovascular treatment. This study aimed to compare the pathological characteristics of lower extremity arteries in HD and non-HD patients with LEAD by evaluating specimens collected from autopsy and amputation cases.

## Methods

### Study population and patient characteristics

**Figure 1:**
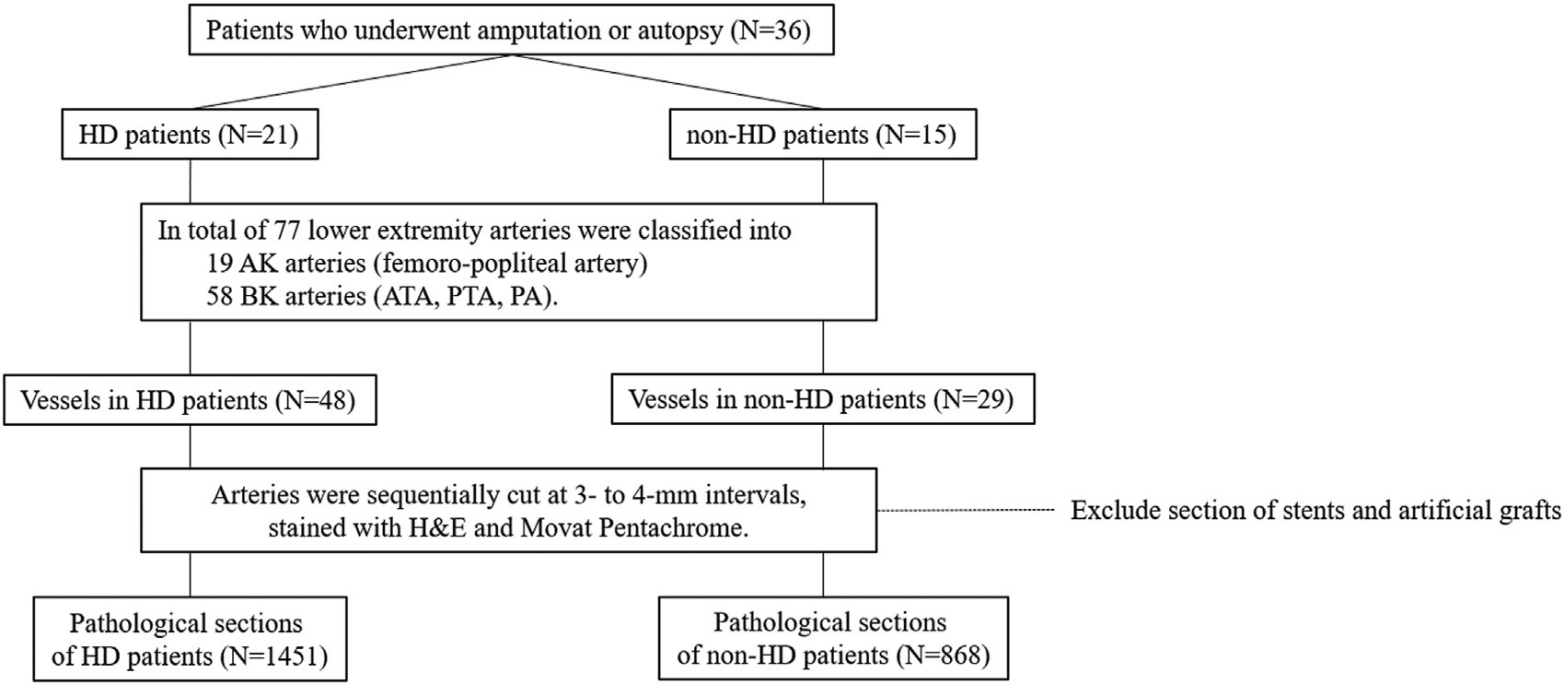
**Study Flow of the Manuscript** AK = above-the-knee; ATA = anterior tibial artery; BK = below-the-knee; HD = hemodialysis; PA = peroneal artery; PTA = posterior tibial artery.

A total of 36 patients with lower extremity amputation due to CLTI or acute limb ischemia (ALI) and autopsy subjects who died from various causes were enrolled in the current study. ALI was defined as an ischemic limb with acute onset to progressive exacerbation,^18^ excluding acute progression of CLTI. The patients were listed in the "CV Hills pathology registry of LEAD patients" from November 2019 to December 2021. The registry involved 14 centers in Japan and included patients with symptomatic LEAD in Rutherford Categories 3 (severe claudication) to 6 (ulceration or gangrene). We histologically evaluated all 36 patients (39 limbs) who were divided into 2 patient groups: HD (n = 21) and non-HD (n = 15), (Figure 1).

This study was approved by the Ethics Review Committee of Tokai University (No. 19R289).

### Pathological evaluation of the lower extremity artery

Lower extremity arteries were trimmed from the legs and classified into 2 groups based on X-ray and gross findings: femoropopliteal arteries were classified as above-the-knee (AK) arteries, while anterior tibial arteries, posterior tibial arteries, and peroneal arteries were classified as BK arteries. In all, 32 patients had undergone previous revascularization (31 endovascular therapy and 1 bypass). Sections with stents and artificial grafts were excluded from the evaluation. We fixed 77 arteries (19 AK and 58 BK) in 10% formalin, and anatomic location and calcification severity were determined using radiographs. Vessels were further decalcified as necessary. All arteries were cut sequentially in 3- to 4-mm intervals and tissue sections were prepared and stained with hematoxylin and eosin, and Movat pentachrome as previously reported.^19^ Morphometric analysis and calcification measurements were performed in the scanned sections using ZEN software (ZEISS) as previously demonstrated.^16^ In brief, the percent stenosis and circumferential angle (arc) of intimal and medial calcification were measured. The sections stained with hematoxylin and eosin were used to evaluate calcification severity, and the Movat pentachrome stain was used to determine the exact media location (internal and external elastic laminae). Medial calcification was defined as primary calcification within the media and did not include intimal calcification extending to the medial wall. The internal and external laminae, the inner and outer border of the media, respectively, were carefully examined especially in sections stained with Movat pentachrome stain.

### Classification of atherosclerotic plaque and calcification

Intimal plaques were classified following the modified American Heart Association classification.^12^^,^^16^^,^^17^^,^^20^^,^^21^ In brief, adaptive intimal thickening was defined as the spontaneous accumulation of smooth muscle cells (SMCs) within the proteoglycan collagen matrix, with the absence of lipid or macrophage foam cell infiltration. Pathological intimal thickening (PIT) was defined as the lipid pool that indicates the presence of apoptotic SMCs. Fibroatheroma was characterized by the presence of macrophages around a necrotic core, with a dense fibrous cap. Thin-cap fibroatheroma was identified as a necrotic core covered by a thin fibrous cap. Plaque rupture consists of a necrotic core and an overlying disrupted thin fibrous cap. Fibrous plaque was characterized by collagen-rich neointimal tissue with a few SMCs but no lipid pool or necrotic core. Fibrocalcific plaque was defined as neointimal growth with calcified lesions, and calcified nodule was defined as calcified lesions erupting into the vessel lumen with thrombi on the surface.

Adaptive intimal thickening, fibrous plaque and PIT were classified as nonadvanced atherosclerotic lesions, whereas fibroatheroma, plaque rupture, thin-cap fibroatheroma, fibrocalcific plaque, and calcified nodule were classified as advanced atherosclerotic lesions. Calcification with a circumferential angle (arc) ≥180° was defined as severe calcification. Bone formation was also evaluated with the arc measurement in the media and intima similar to calcification. Bone formation with a circumferential angle ≥90° was defined as severe bone formation.

### Statistical analysis

Results for normally distributed continuous variables are expressed as mean ± SD. Nonnormally distributed variables are expressed as median IQR. Categorical data were analyzed using the Chi-squared test or the Fisher test. Variables with nonparametric distributions were compared using the Kruskal–Wallis test. The generalized estimating equation (GEE) method was used for vessel-level analysis. Continuous variables were tested using the GEE method with log-linked or Poisson models, as appropriate. Categorical data were tested using the ordinal logistic model or tube Fisher exact test to the GEE method. In the multivariable analysis with logistic regression performed to examine predictors of medial calcification, variables with *P* < 0.05 on univariable analysis were used. When clinically similar variables remained, we selected variables that were considered more clinically relevant. JMP version 16.0.0 (SAS Institute) and GraphPad Prism version 7.03 (GraphPad), SPSS software version 27 (IBM Corporation) were used for statistical analysis.

## Results

### Patient and lesion characteristics

This study evaluated 2,319 sections (HD group, n = 1,451; non-HD group, n = 868) from 77 lower extremity arteries (HD group, n = 48; non-HD group, n = 29). Patient characteristics (eg, age, gender, major cardiovascular risk factors, and medication) were similar between the groups (Table 1). ALI prevalence was higher in the non-HD group (HD vs non-HD group, 4.8% vs 33.3%; *P* = 0.01). More than 90% of patients had CLTI with Rutherford category ≥ IV, resulting in majority of lower limb amputations in both groups. The causes of death in the 5 autopsy cases were 2 heart failures, 2 sepsis due to lower extremity gangrene, and 1 noncardiovascular death. Serum phosphorus levels were significantly higher in the HD group than in the non-HD group (4.3 [IQR: 3.0-6.4] mg/dL vs 3.2 [IQR: 2.9-3.4] mg/dL, respectively; *P* = 0.03). Serum LDL cholesterol levels were lower in the HD group than the non-HD group (HD vs non-HD group, 59.0 [IQR: 48.0-106] mg/dL vs 82.0 [IQR: 55.3-103] mg/dL, respectively; *P* = 0.5), with low statin usage (HD vs nonHD group, 23.8% vs 46.7%, respectively; *P* = 0.20). The median HD duration was 7.5 years (IQR, 5.3-11.0). Vessel characteristics such as distribution, percent stenosis, and the prevalence of chronic total occlusion were similar between the 2 groups (Table 2).

**Table 1:** Comparison of Patient Characteristics Between HD and Non-HD Groups

	HD (N = 21)	Non-HD (N = 15)	*P* Value
Age (y)	77 (59.5-81.5)	76 (70-79)	0.80
Male	13 (61.9)	10 (66.7)	0.80
Body mass index (kg/m^2^)	18.2 (17.2-23.7)	18.3 (16.5-23.5)	0.80
Hypertension	17 (81.0)	10 (66.7)	0.30
Diabetes	13 (61.9)	6 (40.0)	0.20
Dyslipidemia	8 (38.1)	5 (33.3)	0.80
Smoking	7 (33.3)	5 (33.3)	1.00
Coronary artery disease	13 (61.9)	5 (33.3)	0.09
History of PCI	8 (38.1)	3(20.0)	0.20
History of CABG	3 (14.2)	0 (0)	
Mild stenosis diagnosed with Coronary angiography	2 (9.5)	3 (13.3)	
Atrial fibrillation	1 (4.8)	4 (26.7)	0.06
Chronic kidney disease	21 (100)	5 (33.3)	<0.0001
Acute limb ischemia	1 (4.8)	5 (33.3)	0.02
Rutherford classification			
≥Ⅳ	21 (100)	14 (93.3)	0.20
Amputation or autopsy			
Amputation	19 (90.5)	12 (80.0)	0.40
Autopsy	2 (9.5)	3 (20.0)	
Laboratory data			
Ca (mg/dL)	8.5 (7.9-9.1)	8.4 (8.2-8.9)	0.90
P (mg/dL)	4.3 (3.0-6.4)	3.2 (2.9-3.4)	0.03
PTH-INTACT (pg/ml)	138 (81.0-183)	–	–
TG (mg/dL)	91.5 (70.8-125)	84 (61.6-115)	0.50
HDL-C (mg/dL)	44 (38.0-58.0)	39.5 (32.8-60.3)	0.70
LDL-C (mg/dL)	59.0 (48.0-106)	82.0 (55.3-103)	0.50
HbA1c (%)	6.0 (4.9-6.4)	6.5 (5.6-7.1)	0.20
Medication			
Antiplatelet use	19 (90.5)	13 (86.7)	0.70
Anticoagulant use	2 (9.5)	5 (33.3)	0.08
Statin use	5 (23.8)	7 (46.7)	0.20

Values are median (IQR) or n (%).

Ca = calcium; CABG = coronary artery bypass graft; CAG = coronary angiography; HD = hemodialysis; HDL-C = high density lipoprotein cholesterol; LDL-C = low density lipoprotein cholesterol; P = phosphorus; PCI = percutaneous coronary intervention; PTH = parathyroid hormone; TG = triglyceride.

**Table 2:** Comparison of Vessel Characteristics Between HD and Non-HD Groups

	All (N = 77)	HD (N = 48)	Non-HD (N = 29)	*P* Value
Lesion distribution				
AK	19 (24.7)	9 (18.8)	10 (34.5)	0.10
BK	58 (75.3)	39 (81.3)	19 (65.5)	
% stenosis (%)	50.0 (33.5-71.4)	50.8 (33.4-76.4)	47.6 (34.6-66.1)	0.50
CTO lesion	27 (35.1)	18 (37.5)	9 (31.0)	0.60

Values are n (%) or median (IQR).

AK = above-the-knee; BK = below-the-knee; CTO = chronic total occlusion; HD = hemodialysis.

### Calcification and bone formation in lower extremity arteries

**Figure 2:**
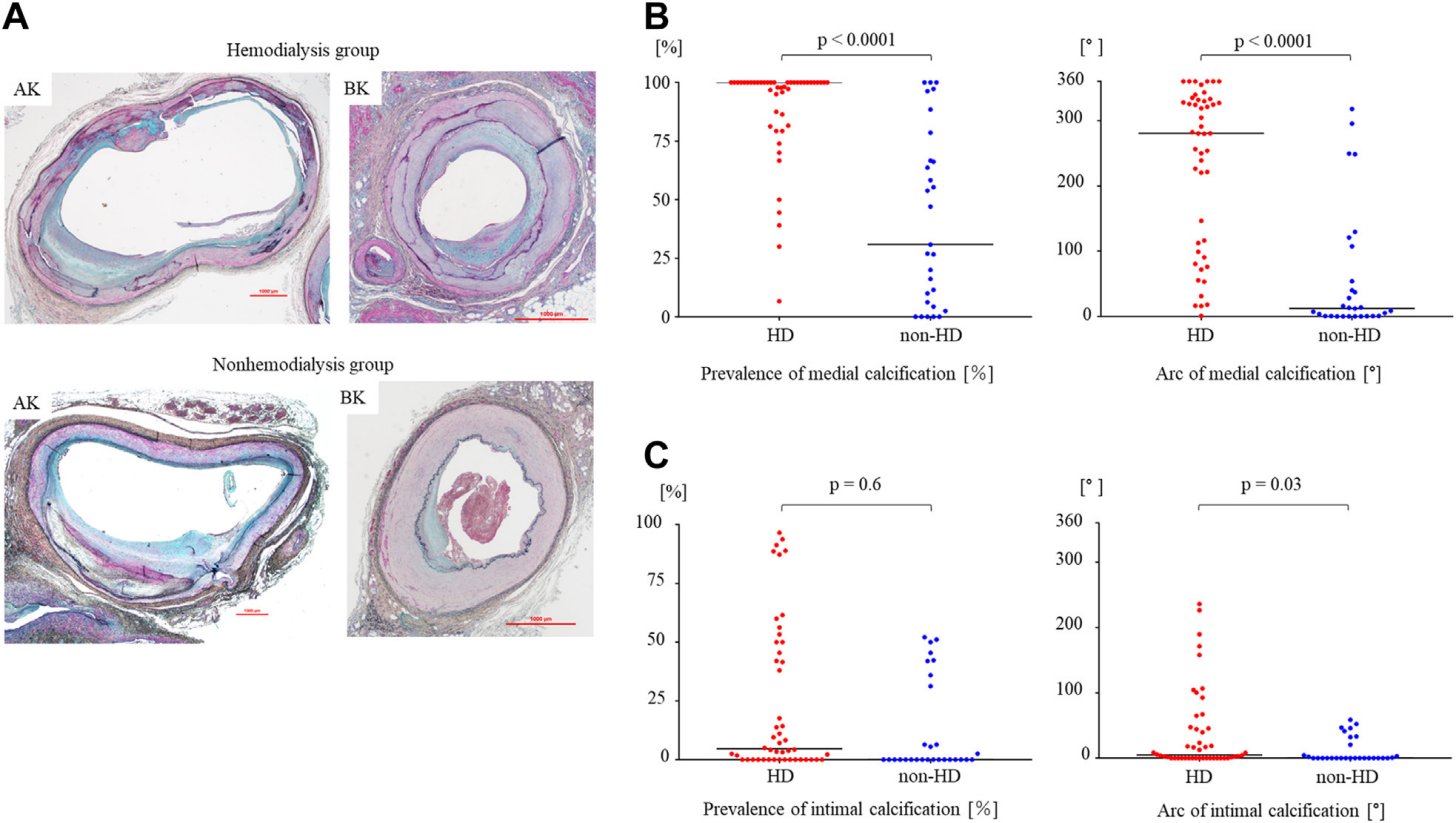
**Comparison of Calcified Lesions and Bone Formation in the** HD **and Non-HD Groups** (A) Representative histopathological images (Movat pentachrome staining) of AK and BK in the hemodialysis HD patients and nonHD patients. Both AK and BK sections demonstrated more extensive medial calcification. Most of the BK lesions in the HD patients were surrounded by circumferential medial calcification. (B) Comparison of medial calcification in HD and non-HD groups. The prevalence and arc of medial calcification were significantly higher in the HD patients than in the non-HD patients. (C) Comparison of intimal calcification in dialysis and nondialysis groups. The arc of intimal calcification was significantly higher in the HD Patients than the non-HD patients. AK = above-the-knee; BK = below-the-knee; HD = hemodialysis.

Figure 2A shows the representative pathological images. Medial calcification was significantly more prevalent in the HD group than the non-HD group (100.0% [IQR: 82.7%-100.0%] vs 30.9% [IQR: 5.3%-72.6%], respectively; *P* < 0.0001). The medial calcification arc was also significantly greater in HD patients (280.5° [IQR: 102.4°-331.2°] vs 12.1° [IQR: 0.6°-80.7°], respectively, *P* < 0.0001) (Figure 2B). On the other hand, the prevalence of intimal calcification was similar between the 2 groups (4.7% [IQR: 0%-48.9%] vs 0% [IQR: 0%-33.6%], respectively; *P* = 0.60). However, the intimal calcification arc was greater in HD patients (4.9° [IQR: 0°-47.0°] vs 0° [IQR: 0°-26.3°], respectively; *P* = 0.03) (Figure 2C).

**Figure 3:**
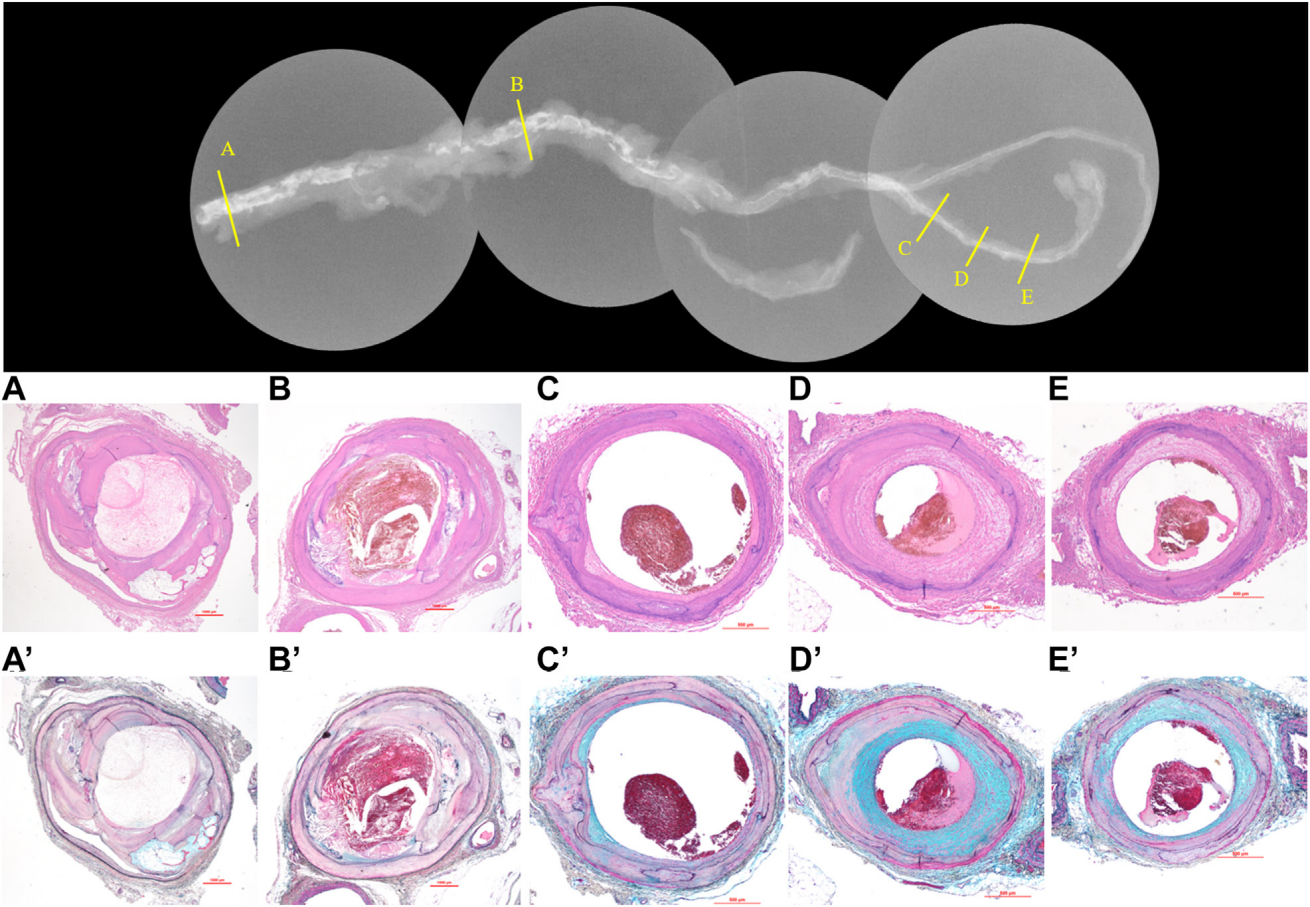
**Histopathological Findings of Above-the-Knee to Below-the-Knee in Patients Undergoing HD With Chronic Limb-Threatening Ischemia** A 77-year-old woman with end-stage renal failure due to diabetic nephropathy and chronic limb-threatening ischemia with Rutherford category V. A left lower extremity above-the-knee amputation was performed. Hematoxylin and eosin-stained images of femoropopliteal arteries (A and B) and peroneal arteries (C-E). Corresponding Movat pentachrome stained images (A′-E′) are shown. The femoropopliteal artery showed sheet calcification of the intima (A and B), with partial bone formation in proximity (A). A ruptured necrotic core in the popliteal artery is present, indicating subocclusion due to acute thrombosis (B). The peroneal artery (C-E) showed extensive medial calcification and fibrous plaque in the intima. Luminal thrombus was observed without any intimal atherosclerotic changes, suggesting distal emboli from plaque rupture in section B. BF = bone formation; IC = intimal calcification; MC = medial calcification; NC = necrotic core.

Medial calcification in BK lesions and intimal calcification in AK lesions were significantly higher in the HD group when the sections were divided into 2 groups according to vessel location (Table 3). HD and non-HD groups showed extremely rare intimal calcification in BK lesions. Medial calcification was found in almost all the sections in BK lesions of HD patients, with severe medial calcification in 73.3% of the sections. Representative histopathological AK to BK findings in HD patients presenting with CLTI leading to lower limb amputation are shown in Figure 3 (Central Illustration).

**Table 3:** Comparison of Severity of Calcification by Site Between HD and Non-HD Groups

	HD	Non-HD	*P* Value
Calcification			
AK (femoro-popliteal)	(n = 9)	(n = 10)	
Prevalence of medial calc (%)	66.7 (34.5-92.1)	21.5 (5.3-59.7)	0.07
Medial calc arc (°)	31.2 (16.1-130)	5.41 (0.6-16.1)	0.08
Prevalence of intimal calc (%)	60 (43.5-88.1)	18.9 (0-46.6)	0.02
Intimal calc arc (°)	92.7 (44.9-147)	17 (0-42.5)	0.001
BK (ATA, PTA, PA)	(n = 39)	(n = 19)	
Prevalence of medial calc (%)	100 (96.8-100)	47.1 (0-96.3)	<0.0001
Medial calc arc (°)	319 (239-335)	15.9 (0.6-129)	0.0005
Prevalence of intimal calc (%)	3.2 (0-13.8)	0 (0-5.6)	0.09
Intimal calc arc (°)	2.4 (0-17.1)	0 (0-3.0)	0.20

Values are median (IQR).

AK = above-the-knee; ATA = anterior tibial artery; BK = below-the-knee; calc = calcification; HD = hemodialysis; PA = peroneal artery; PTA = posterior tibial artery.

**Central Illustration:**
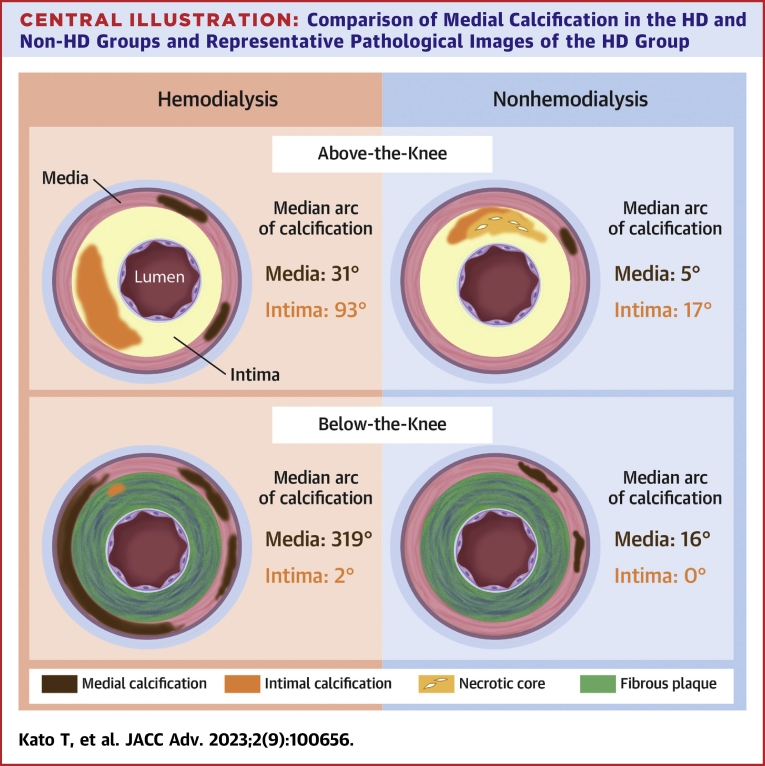
**Comparison of Medial Calcification in the HD and Non-HD Groups and Representative Pathological Images of the HD Group** Representative histopathological images (Movat pentachrome staining) of AK and BK in the HD patients (A and B). Both AK and BK sections demonstrated more extensive medial calcification. Most of the BK lesions in the HD patients were surrounded by circumferential medial calcification. The prevalence and arc of medial calcification were significantly higher in the HD patients than the non-HD patients (C). AK = above-the-knee; BK = below-the-knee; HD = hemodialysis.

Univariable and multivariable analyses were performed to identify risk factors for severe medial calcification in BK lesions (Table 4). Multivariable analysis demonstrated that HD was an important factor associated with severe medial calcification in BK lesions (OR: 16.2; 95% CI: 1.6-167; *P* = 0.02).

**Table 4:** Uni/Multivariable Analyses of Predictors for Severe Medial Calcification

	Univariable Analyses				Multivariable Analyses			
	OR	Lower (95% CI)	Upper (95% CI)	*P* Value	OR	Lower (95% CI)	Upper (95% CI)	*P* Value
Hemodialysis	17.1	4.3	67.7	<0.0001	16.2	1.6	167	0.02
Phosphorus ≥4.6 mg/dL	18.0	2.1	152.5	0.0004	4.0	0.4	43.1	0.30
Diabetes	3.8	1.2	11.5	0.02	1.6	0.2	11.8	0.80
BMI ≥23 kg/m^2^	3.2	1.0	10.5	0.04	9.2	0.6	137	0.10
Age ≥75 y	0.7	0.2	2.0	0.50				
Male	0.7	0.2	2.2	0.60				
Hypertension	2.9	0.9	9.2	0.07				
Coronary artery disease	1.5	0.5	4.9	0.50				
LDL ≥70 mg/dL	0.4	0.1	1.5	0.20				

BMI = body mass index.

**Figure 4:**
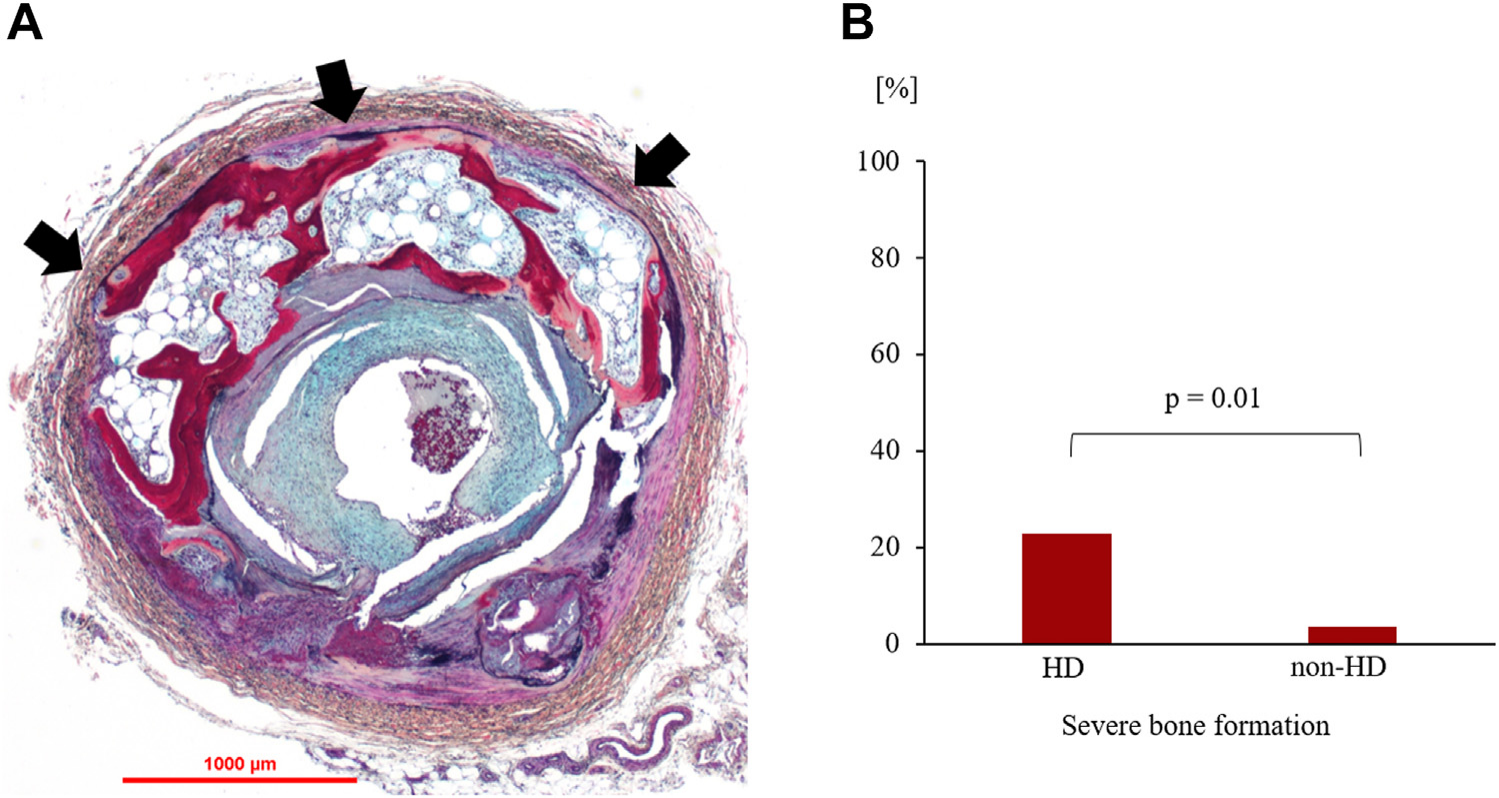
**Comparison of the Prevalence of Severe Bone Formation in HD and Non-HD Groups** (A) A representative case with findings of medial bone formation. A 49-year-old man with end-stage renal failure due to diabetic nephropathy and a 13-year history of HD underwent below-the-knee amputation. Severe medial bone formation was observed proximal to the PTA (arrows). The intima showed fibrous plaque with luminal thrombus, embolized from the proximal above-the-knee lesion. (B) Lower extremity arteries with severe bone formation (circumferential angle ≥90°) were significantly more common in the HD group. HD = hemodialysis; PTA = posterior tibial artery.

The number of lower extremity arteries with medial or intimal bone formation was 15 (31.3%) and 5 (17.2%) in the HD and non-HD groups, respectively (Table 5). Bone marrow was found in 76% (100 of 132 sections) of the sections with bone formation. Severe bone formation (combined medial and intimal) was more common in the HD group than the non-HD group (22.9% vs 3.5%, respectively; *P* = 0.01) (Figure 4).

**Table 5:** Comparison of Bone Formation by Site Between HD and Non-HD Groups

	HD (N = 48)	non-HD (N = 29)	*P* Value
Bone formation			
All (Medial and/or Intimal)	15 (31.3)	5 (17.2)	0.20
Medial	7 (14.6)	2 (6.9)	0.30
Intimal	12 (25.0)	4 (13.8)	0.20

Values are n (%).

HD = hemodialysis.

### Intimal plaque classification in lower extremities

**Figure 5:**
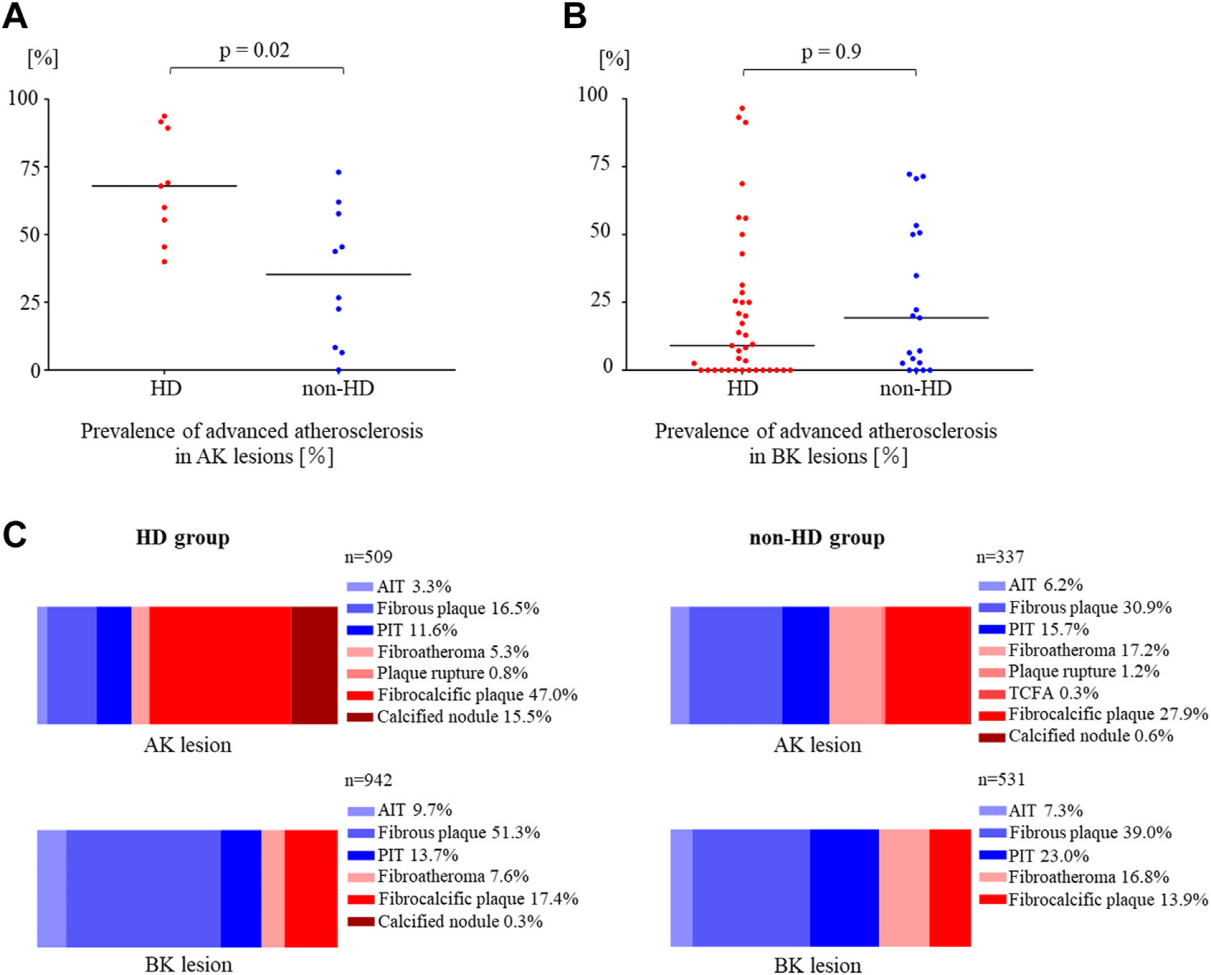
**Comparison of Atherosclerotic Lesions in HD and Non-HD Groups** The prevalence of intimal atherosclerosis was common in the HD group when restricted to AK lesions (A), whereas no significant difference in the prevalence of intimal atherosclerosis was found between the HD and non-HD groups when restricted to BK lesions (B). (C) Comparison of intimal plaque types by site in HD and non-HD groups. In the HD group, the fibrocalcific plaque was the most common plaque type, followed by fibrous plaque and calcified nodule. In the NonHD group fibrous plaques were the most common AK lesion. Fibrous plaques were the most common plaque type of BK lesion in both groups, and advanced atherosclerosis was less common. AIT = adaptive intimal thickening; AK = above-the-knee; BK = below-the-knee; HD = hemodialysis; PIT = pathological intimal thickening; TCFA = thin-cap fibroatheroma.

The prevalence of advanced intimal atherosclerosis in AK lesions was significantly higher in the HD group compared to the non-HD group (67.9% [IQR: 50.4%-90.5%] vs 35.2% [IQR: 7.9%-58.8%], respectively; *P* = 0.02) (Figure 5A). The prevalence of advanced intimal atherosclerosis in BK lesions was similar in the 2 groups (HD group, 9.1% [IQR: 0%-28.6%] vs non-HD group 19.2% [IQR: 2.6%-50.6%], respectively; *P* = 0.9) (Figure 5B).

In AK lesions, in the HD group, fibrocalcific plaque was the most common plaque type (47.0%), followed by fibrous plaque (16.5%) and calcified nodule (15.5%); and in the non-HD group, fibrous plaque (30.9%) was the most common followed by fibrocalcific plaque (27.9%).

In BK lesions of both the HD and non-HD groups, fibrous plaque was the most common plaque type (51.3% vs 39.0%) (Figure 5C).

### Thrombotic lesions

**Figure 6:**
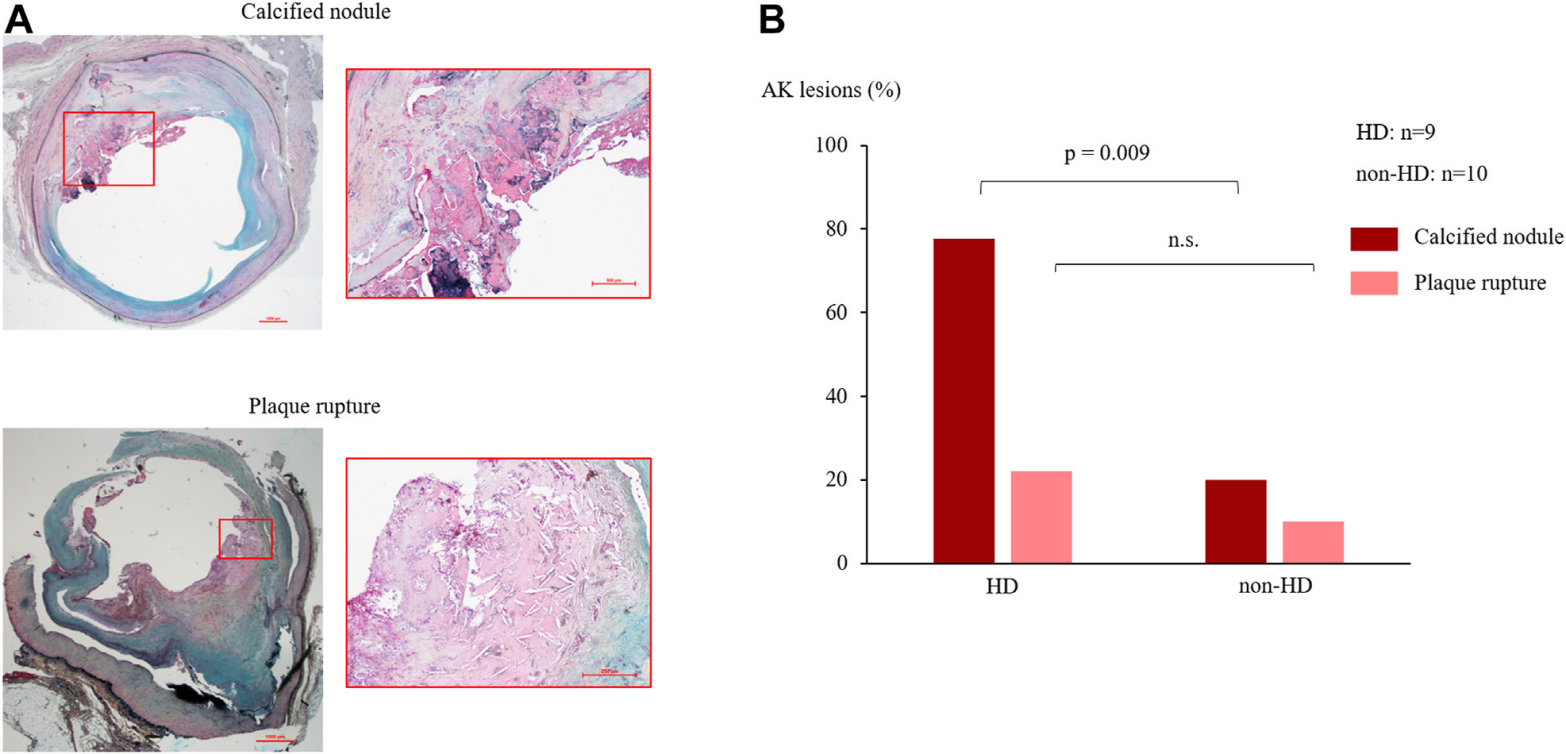
**Comparison of the Incidence of Calcified Nodules and Plaque Rupture Resulting in Acute Thrombotic Events in Above-the-Knee Lesions in the HD and Non-HD Groups** (A) Representative histopathological images (Movat pentachrome staining) of calcified nodule and plaque rupture in AK lesions. (B) The prevalence of lesions with calcified nodules in AK lesions was greater in the HD group than the Non-HD group. Moreover, the prevalence of lesions with plaque rupture was similar in both groups. AK = above-the-knee; HD = hemodialysis.

All the thrombotic lesions (calcified nodule or plaque rupture) were observed in the AK group, except for 1 lesion that showed a calcified nodule in anterior tibial artery. The prevalence of vessels with calcified nodules in AK lesions was more common in the HD group than the non-HD group (77.8% vs 20.0%, respectively; *P* = 0.009). The prevalence of lesions with plaque rupture was similar in both groups (22.2% vs 10.0%, respectively; *P* = 0.46) (Figure 6).

## Discussion

The increasing number of patients undergoing HD with LEAD has resulted in more amputations and deaths due to CLTI; however, the reasons for poor clinical outcomes in patients on HD have not been well evaluated. This study aimed to investigate the LEAD pathologic features in HD patients collected at autopsy and from amputated legs. The main findings were as follows: 1) the arc of intimal and medial calcification were significantly higher in the HD patients than the non-HD patients; 2) HD was an important factor associated with severe medial calcification in BK lesions; 3) severe bone formation was more common in the HD patients; 4) the HD patients had more advanced intimal atherosclerotic AK lesions than the non-HD patients but the groups had similar plaque characteristics in BK lesions; and 5) calcified nodules were more common in the HD patients compared to non-HD patients with a similar prevalence of plaque rupture.

### Medial/intimal calcification in lower extremity arteries in HD patients

A higher degree of calcification in the lower extremities is a common cause of poor prognosis in patients undergoing HD. Additionally, medial calcification as seen in LEAD is rarely seen in coronary arteries.^22^ Therefore, understanding its prevalence and characteristics in patients undergoing HD is critical to improving their clinical outcomes. Previous pathological studies of lower extremities have demonstrated the association of age, diabetes, and chronic kidney disease with arterial calcification.^23^^,^^24^ However, reports on calcification of lower extremity arteries did not differentiate medial and intimal calcification. Ohtake et al,^10^ for example, used multidetector-row CT to investigate the degree of lower limb arterial calcification in HD patients. However, they did not distinguish between medial and intimal calcification.^10^ Another study on HD patients identified the linear rail track type as medial calcification, and irregular and patchy distribution type as intimal calcification based on X-rays, which may be not precise enough for identifying the calcification distribution. In addition, arterial calcification was only analyzed in limited locations (pelvis and thigh), not including BK arteries.^9^

The current study evaluated all the histopathological specimens collected from the lower limb arteries, resulting in the most accurate characterization to date in HD patients with LEAD. The results showed that the degree of medial and intimal calcification was significantly higher in HD patients than in non-HD patients. The prevalence and arc of medial calcification in BK lesions were particularly striking, and, surprisingly, a median circumferential calcification of more than 300° was observed in HD patients. Medial calcification was more pronounced in the HD group even though the Rutherford class was comparable in both groups. The study suggests that intimal and medial calcification would be a likely cause of worse clinical outcomes in HD patients.

Medial calcification is reported to be associated with cardiovascular mortality and lower extremity amputation due to loss of arterial wall elasticity and reduced perfusion to peripheral tissues.^24^, ^25^, ^26^ This suggests that medial calcification could affect the prognosis of CLTI patients. Reports suggest that medial calcification is a result of various processes (eg, inflammation, apoptosis, Ca/P homeostasis, and extracellular matrix organization); however, the mechanisms of its progression are still unclear.^22^^,^^27^ Another study also demonstrated significant medial calcification in patients without common cardiovascular risk factors.^28^

The present study supports findings of HD as an important factor associated with severe medial calcification in BK lesions. Furthermore, significantly higher serum phosphorus levels in the HD group suggest HD-induced electrolyte abnormalities may have contributed to medial calcification. These results highlight the importance of dialysis quality, which is dependent upon the dialysis membrane and fluids, to maintain optimal serum phosphorus levels. Further studies are therefore needed to reveal the mechanisms of the medial calcification progression.

### Bone formation in HD patients

Previous pathologic evaluation demonstrated that bone formation was observed in 6% of the lower extremity arteries of amputated limbs.^24^ Age and diabetes mellitus were associated with bone formation, whereas renal failure was not. However, the prevalence of bone formation may have been underreported because not all the serial sections were evaluated in the study. There were few sections with bone formation (5.7%; 132 in 2,319 sections), whereas the current study demonstrated bone formation in 20 lower extremity arteries (26%). Evaluating all the serial sections of the artery is believed to be the most accurate method to determine the prevalence of bone formation. The higher prevalence of bone formation in lower extremity arteries could be a cause of poor prognosis in patients undergoing HD. Recent histological studies have shown the effectiveness of optical coherence tomography in detecting calcification and bone formation in the lower extremities.^29^ Further observational studies are needed to evaluate the association of bone formation and prognosis, such as amputation.

### Intimal plaque classification in lower extremities

Several studies have investigated the pathological characteristics of intimal plaque in lower limb arteries. Torii et al ^16^ and Narula et al ^17^ performed a detailed pathological evaluation of LEAD patients. However, studies have not focused on HD patients. In the current study, the intimal plaque type of all tissue specimens from HD and non-HD patients was assessed following the Modified American Heart Association classification. Intimal calcification was significantly more common in the HD patients than the nonHD patients in AK lesions. Furthermore, advanced atherosclerotic changes (eg, fibrocalcific plaque and calcified nodule in AK lesions) were more frequent in the HD group. Moreover, fibrous plaques were most frequently observed in the BK lesions of HD patients compared to non-HD patients with fewer advanced atherosclerotic changes. Atherosclerotic changes in lower extremity arteries were more pronounced in AK lesions, which was in line with previous pathological studies.^16^^,^^17^ Ablation with debulking devices such as the Jetstream atherectomy device (Boston Scientific), orbital atherectomy (Diamondback 360, Cardiovascular Systems Inc) or intravascular lithotripsy (Shockwave medical) might be effective for intimal calcification such as fibrocalcific plaques and calcified nodules, which were relatively common in the HD group. Conversely, as the degree of atherosclerosis in BK lesions was not as advanced as expected, newer treatment devices for endovascular treatment of BK lesions in HD patients will be needed to improve clinical outcomes.

### Study limitations

First, the number of subjects evaluated was relatively small, and the number and length of vessels trimmed varied. However, the number of subjects may be enough to characterize the pathological differences between medial and intimal calcification in HD and non-HD patients because all the serial sections of artery were evaluated in the study with detailed clinical data (eg, serum P/Ca levels). Second, the prevalence of coronary artery disease in the current study may be underdiagnosed as coronary angiography or coronary CT was not performed in all the cases. Third, detailed laboratory parameters including patient coagulation parameters were not available.

## Conclusions

HD patients had a higher degree of medial and intimal calcification compared with non-HD patients, and the difference was greatest in medial calcification of BK lesions. Further research is needed to reveal the mechanism and prevention of calcification in HD patients and to improve their prognosis.

PERSPECTIVES **COMPETENCY IN MEDICAL KNOWLEDGE:** Pathological features of LEAD in HD patients with poor prognosis were examined. The degree of intimal and medial calcification was significantly higher in the HD patients compared to the non-HD patients. Especially, medial calcification in BK lesions were considered to be the most important feature in HD patients. As for plaque characteristics of the intima, HD patients had significantly higher atherosclerotic changes in AK lesions compared to non-HD patients, but similar levels in BK lesions. Treatment strategies should be considered according to the lesion.

**TRANSLATIONAL OUTLOOK:** Ablation with debulking devices might be effective for intimal calcification such as fibrocalcific plaques and calcified nodules, which were relatively common among AK lesions in the HD group. In BK lesions of HD patients with significant medial calcification, thorough serum phosphorus level control and antithrombotic therapy are considered important. Novel treatment strategies for medial calcification are expected to be developed to improve the prognosis of HD patients with lower extremity arterial disease.

## Funding support and author disclosures

This work was supported by 10.13039/501100001691JSPS KAKENHI (grant number JP20K17097). Dr Torii has received research grants from Abbott Vascular Japan, Boston Scientific Japan, and Medtronic; and has received honoraria from Boston Scientific Japan. Dr Nakama is a consultant for Becton Dickinson, Boston Scientific, COOK Medical, Medtronic, Century Medical Inc, Cordis, NIPRO, and OrbusNeich. Dr Nakazawa is a consultant for Boston Scientific, Abbott Vascular, Terumo Corp, and Japan Medical Device Technology Co, Ltd, ZAIKEN; and has received research grants from Boston Scientific, Abbott Vascular, Terumo Corp., and Japan Medical Device Technology Co, Ltd. All other authors have reported that they have no relationships relevant to the contents of this paper to disclose.
